# Hem1 is essential for ruffled border formation in osteoclasts and efficient bone resorption

**DOI:** 10.1038/s41598-024-58110-x

**Published:** 2024-04-06

**Authors:** Eugenie Werbenko, David J. J. de Gorter, Simon Kleimann, Denise Beckmann, Vanessa Waltereit-Kracke, Julia Reinhardt, Fabienne Geers, Peter Paruzel, Uwe Hansen, Thomas Pap, Theresia E. B. Stradal, Berno Dankbar

**Affiliations:** 1 Institute of Musculoskeletal Medicine, University Hospital Muenster, Albert-Schweitzer-Campus 1, Building D3, 48149 Muenster, Germany; 2 Department of Cell Biology, Helmholtz Centre for Infection Research (HZI), Braunschweig, Germany

**Keywords:** Hem1, Wave, Osteoclast, Ruffled border, Cell biology, Cytoskeleton

## Abstract

Bone resorption is highly dependent on the dynamic rearrangement of the osteoclast actin cytoskeleton to allow formation of actin rings and a functional ruffled border. Hem1 is a hematopoietic-specific subunit of the WAVE-complex which regulates actin polymerization and is crucial for lamellipodia formation in hematopoietic cell types. However, its role in osteoclast differentiation and function is still unknown. Here, we show that although the absence of Hem1 promotes osteoclastogenesis, the ability of Hem1^-/-^ osteoclasts to degrade bone was severely impaired. Global as well as osteoclast-specific deletion of *Hem1 *in vivo revealed increased femoral trabecular bone mass despite elevated numbers of osteoclasts in vivo. We found that the resorption defect derived from the morphological distortion of the actin-rich sealing zone and ruffled border deformation in Hem1-deficient osteoclasts leading to impaired vesicle transport and increased intracellular acidification. Collectively, our data identify Hem1 as a yet unknown key player in bone remodeling by regulating ruffled border formation and consequently the resorptive capacity of osteoclasts.

## Introduction

Throughout life the skeleton is constantly remodeled through bone formation by osteoblasts and bone resorption by osteoclasts. A shift in either direction leads to an imbalance in bone turnover and forms the basis for skeletal disorders. Many skeletal diseases, including osteoporosis and rheumatoid arthritis, are associated with excessive bone erosion caused by osteoclasts. On the other hand, defects in osteoclast differentiation or function manifest in excessive bone formation termed osteopetrosis^1,2^.

Osteoclasts are large multinucleated cells that possess the unique ability to degrade bone matrix. In order to exert their resorptive function osteoclasts polarize towards bone which involves an extreme re-organization of their cytoskeleton^3,4^. Attachment to bone induces the formation of predominantly αvß3-integrin-mediated adhesion structures, called podosomes, that are organized as an actin-rich ring forming the sealing zone^4–7^. This allows a confinement of the resorptive compartment, separating it from the extracellular space and enabling acidification and bone degradation. Within this compartment, the osteoclast membrane in close contact to the bone surface expands into the ruffled border by fusion of secretory vesicles with the bone-apposed osteoclast membrane resulting in a highly convoluted, finger-like membrane^8–13^. However, the exact mechanism of how the ruffled border is established is still not fully understood. Of importance, several mutations in genes encoding proteins involved in ruffled border formation as well as lysosomal trafficking have been implicated in impaired osteoclast-mediated bone resorption, even though osteoclastogenesis itself was not affected^14^.

Dynamic remodeling of the actin cytoskeleton is required for mechanical force generation and is essential for all types of cell migration, cell adhesion as well as endo- and exocytosis. Protrusions and invaginations of the cell membrane are shaped by actin filaments growing at their barbed ends and pushing the membrane forward. Filamentous actin (F-actin) is born by nucleation and is initiated by actin nucleating proteins, the most prominent being the actin-related proteins-2/3 (Arp2/3) complex. Members of the Wiskott-Aldrich syndrome protein (WASP) family are the main mediators of integration of incoming signals upstream from Rho family of small GTPases and downstream activating the Arp2/3 complex eventually leading to actin assembly. WASP functions downstream of the Rho GTPase Cdc42 and mediates the formation of podosomes and actin assembly required for endocytosis, whereas Rac GTPases activate the WASP-family verprolin homologous protein (WAVE) regulatory complex (WRC), required for the formation of lamellipodia^15^.

In this regard, Hem1 is an adaptor protein and the only subunit of the pentameric WRC, whose expression is limited to cells of hematopoietic lineage^16,17^. Several studies implicated Hem1 and the WRC as important regulators of branched actin and their absence resulted in numerous defects in the formation of cell-specific structures. It was shown that interference with WRC expression in adherent cells abrogated lamellipodia and ruffle formation and diminished cell migration^18–20^. In addition, WRC function has been found to be required for interactions of T-cells with antigen presenting cells and T-cell activation^21–23^. Loss of Hem1 in mouse models inhibits actin polymerization, cell polarization and chemotaxis in lymphocytes and neutrophils, impairs B- and T-cell development and interferes with adhesion dynamics and phagocytosis in macrophages, all processes, that are highly dependent on actin rearrangement^17,24,25^. More recently, loss-of-function mutations in the human *NCKAP1L* gene (encoding Hem1) were associated with severe autoimmunity due to aberrant T and B cell function resembling the defects observed in Hem1-deficient mouse models^26,27^. While the role of the Arp2/3 complex and WASP has long been recognized^28,29^, the WRC has only recently been linked to osteoclast development. Although Wang et al. have shown that the absence of Hem1 in mice leads to osteopetrosis due to a defect in osteoclast fusion and resorption^30^, the mechanism by which Hem1 is involved in the development and activity of osteoclasts still needs to be further elucidated.

In this study, we demonstrate that Hem1-deficient mice develop a severe osteopetrosis-like phenotype caused by osteoclast dysfunction, due to a failure in podosome organization and establishing a functional actin ring. Moreover, *Hem1*^*-/-*^ osteoclasts lack the characteristic membrane foldings that are necessary for lysosomal secretion and for uptake of degraded matrix. Thus, our results identified a so far unknown regulator of ruffled border formation in osteoclasts.

## Results

### *Hem1*-deficiency leads to osteopetrosis-like phenotype

**Figure 1:**
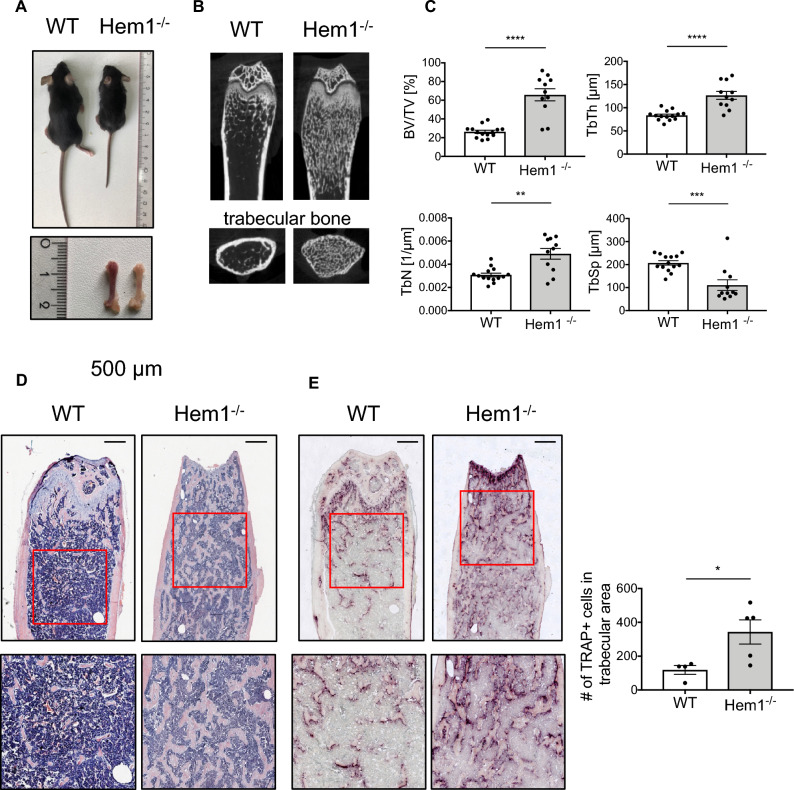
Hem1-deficiency leads to osteopetrosis-like phenotype. (**A**) Hem1^-/-^ mice display signs of growth retardation and reduced femoral length. (**B**) Representative µCT cross sections of distal femora of WT and Hem1^-/-^ animals. (**C**) Statistical analysis of trabecular bone volume to tissue volume (BV/TV,) trabecular thickness (Tb.Th.), trabecular number (Tb.N.) and trabecular separation (Tb.Sp.) of trabecular bone from 8–12 weeks old WT and Hem1^-/-^ male mice. **** *P* < 0.0001 (BV/TV, Tb.Th.), ** = *P* 0.0062 (Tb.N.), *** *P* = 0.0005 (Tb.Sp.), two-tailed Mann–Whitney *U* test. (**D**) Representative H&E staining of distal femora from WT and Hem1^-/-^ mice, red box represents magnified area. (**E**) Left: Representative TRAP staining of distal femora from WT and Hem1^-/-^ mice, red box represents magnified area. Right: Quantification of TRAP-positive osteoclasts within trabecular region from distal femora of WT and Hem1^-/-^ mice. * *P* = 0.0317, two-tailed Mann–Whitney *U* test. All data presented as mean ± SEM.

Mice lacking Hem1 showed a growth delay and reduced femoral length (Fig. 1A), which is in accordance with previous studies^17,30^. Microtomography (µCT) analysis of distal femora revealed that Hem1^-/-^ mice develop a high bone mass phenotype (Fig. 1B). Compared to wild type (WT) and *Hem*^+*/-*^ littermates of the same sex and age, *Hem1*^*-/-*^ mice displayed highly increased trabecular BV/TV ratio by about 150%, elevated trabecular number and thickness by 60% and 52%, respectively, while trabecular separation was reduced by 88% (Fig. 1C, Fig. S1). In line with this observation, H&E staining of WT and *Hem1*^*-/-*^ mice showed that *Hem1* loss leads to a striking increase in trabecular bone mass (Fig. 1D). Interestingly, despite increased trabecular bone mass Tartrate Resistant Acid Phosphatase (TRAP) staining of femoral sections from *Hem1*-deleted mice revealed a 2.9-fold increase in osteoclast numbers compared to WT mice (Fig. 1E).

### *Hem1* deficiency promotes osteoclastogenesis in vitro but reduces resorptive capacity

**Figure 2:**
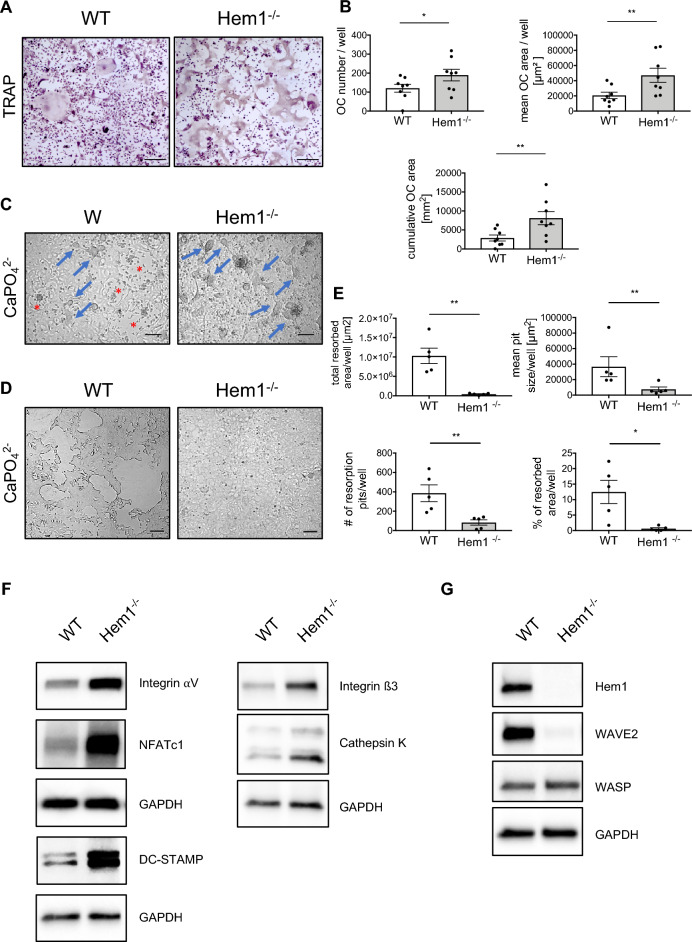
Hem1-deficiency promotes osteoclastogenesis but inhibits resorptive capacity. (**A**) *In-vitro* osteoclast differentiation assay of WT and Hem1^-/-^ osteoclasts visualized by tartrate resistant acid phosphatase (TRAP) staining. Representative images for both genotypes are shown, scale bar 200 µm. (**B**) Quantification of TRAP-assay of WT and Hem1^-/-^ shown in (**A**). * *P* = 0.0234, ** *P* = 0.0078, two-tailed Wilcoxon matched-pairs signed rank test. (**C**) Representative images of WT and Hem1^-/-^ osteoclasts cultured on calcium phosphate (CaPO_4_^2-^; CaP plates) before lysis. Osteoclasts indicated with blue arrows, resorption pits with red asterisk. Scale bar 100 µm. (**D**) Representative images of resorption pit formation of WT and Hem1-/- osteoclasts using calcium phosphate on CaP-coated substrate. Scale bar 100 µm. (**E**) Quantification of (D): total resorbed area/well, number of resorption pits/well, mean pit size/well and percentage of resorbed area/well. ** *P* = 0.0079, * *P* = 0.0159, two-tailed Mann–Whitney *U* test. All data presented as mean ± SEM. (**F**) Immunoblotting of osteoclast markers from WT and Hem1^-/-^ osteoclast lysates, representative images from n = 4 independent experiments. (**G**) Immunoblotting of Hem1, WAVE2 and WASP from WT and Hem1^-/-^ osteoclast lysates, representative images from n = 5 independent experiments.

Increased bone mineral content can arise from increased osteoblast numbers or activity, from decreased osteoclast numbers due to stalled osteoclastogenesis or from impairment of the osteoclasts ability to resorb bone matrix. Since Hem1 is exclusively expressed in cells of hematopoietic origin, we investigated in more detail the impact of *Hem1-*deficiency on osteoclast differentiation in vitro. To this end, bone marrow-derived cells were cultured in the presence of M-CSF and RANKL and osteoclast formation and activity was assessed. As observed in vivo, the absence of *Hem1* led to more and also larger osteoclasts by 50% and 128%, respectively (Fig. 2A,B). Moreover, *Hem1*^*-/-*^ osteoclasts lack the characteristic circular cell morphology of in vitro generated mature osteoclast but rather display an irregular shape (Fig. 2A) indicating altered cytoskeletal organization similar as observed in dendritic cells and macrophages lacking *Hem1*^25,31^.

We performed functional assays to assess the resorption ability of *Hem1*^*-/-*^ osteoclasts. In principle, WT and *Hem1*^*-/-*^ bone marrow-derived cells were able to differentiate into osteoclasts on CaPO_4_^2-^–coated substrate in vitro (Fig. 2C). Strikingly, *Hem1*-deficient osteoclasts hardly formed any pits (Fig. 2D), resulting in a more than 4.5-fold decrease in the number of resorption pits, accompanied by an almost complete disappearance of resorption (Fig. 2E).

We next examined whether expression of important osteoclast differentiation markers was altered during RANKL-induced osteoclast differentiation. In line with the promoted osteoclastogenesis in vivo and in vitro, osteoclasts lacking *Hem1* expressed higher protein levels of NFATc1, a master transcription regulator of osteoclasts^32^, and DC-STAMP, essential for osteoclast fusion^33^ (Fig. 2F). Expression levels of integrin αV and integrin ß3, both involved in osteoclast adhesion, migration, actin ring formation and resorption^5,34–36^, were also increased in *Hem1*-deficient osteoclasts. Moreover, expression levels of the main bone degrading enzyme Cathepsin K^36–39^, were also elevated in *Hem1*^*-/-*^ osteoclasts (Fig. 2F). As observed in other hematopoietic cell populations^17,25,31^, *Hem1* loss in osteoclasts led to a notable deletion of the Arp2/3 binding subunit WAVE2 (Fig. 2G), indicating a loss of WRC in *Hem1*-deficient osteoclasts. Protein expression of WASP, another Arp2/3 complex activator involved in podosome formation^15^, was largely unaltered in *Hem1*-deficient osteoclasts (Fig. 2G).

Taken together, these data indicate that although *Hem1*-deficiency in mice promotes differentiation of osteoclasts including expression of important osteoclastic factors, their resorptive capacity is strongly impaired resulting in increased in vivo trabecular bone mass.

### Hem1 is essential for sealing zone organization and ruffled border formation in osteoclasts

**Figure 3:**
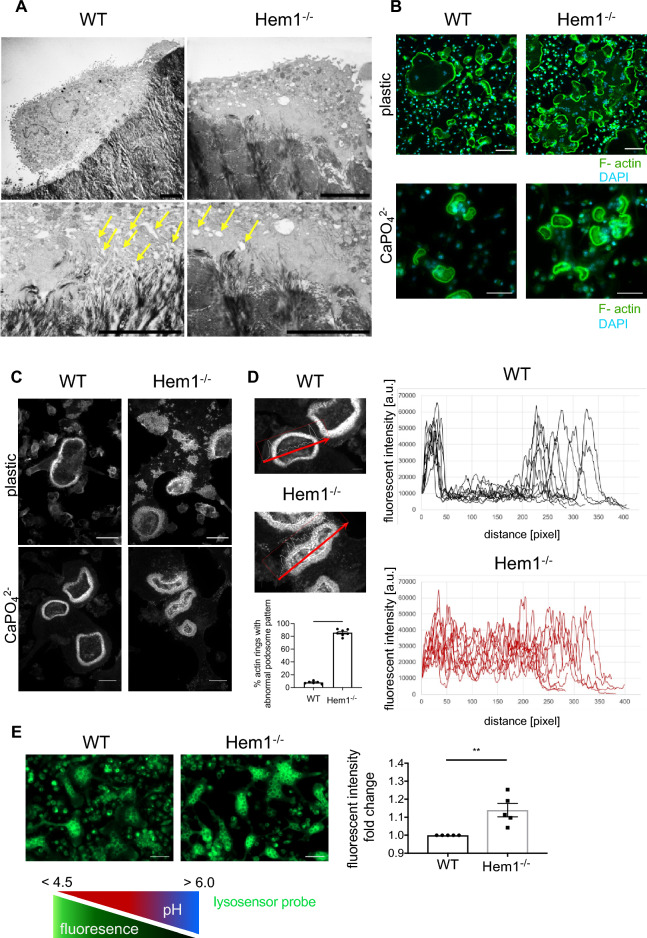
Hem1 is essential for ruffled border formation and actin ring organization. (**A**) Transmission electron microscopy cross-section of WT and Hem1^-/-^ osteoclasts cultured on bovine bone slices. Scale bar 5 µm. Yellow arrows on higher magnification images indicate vesicles close to the basolateral membrane. (**B**) Immunofluorescent visualization of F-actin (green) and nuclei (blue) in osteoclasts cultured on plastic (upper panel) or CaPO_4_^2-^ (lower panel). Scale bar 100 µm (upper panel) 50 µm (lower panel), respectively. Representative images of n = 5 independent experiments. (**C**) Confocal z-stack images showing actin rings in osteoclasts cultured on plastic (upper panel) or CaPO_4_^2-^ (lower panel) visualized by F-actin staining. Scale bar 50 µm. Representative images of n = 3 (upper panel) and n = 4 (lower panel) independent experiments. (**D**) Percentage of disturbed actin rings in OC cultured on CaPO_4_^2-^ and analysis of F-actin intensity throughout the individual actin rings. Representative analysis of n = 4 independent experiments. (**E**) Left: visualization of acidic vesicles (green) in mature WT and Hem1^-/-^ osteoclasts with LysoSensor™ Green DND-189. Scale bar 50 µm. Right: quantification of fluorescent intensity n = 5. ** *P* = 0.0079, Wilcoxon matched-pairs signed rank test. All data presented as mean ± SEM.

Since osteoclasts have a unique cytoskeleton, we further explored the role of Hem1 and the WRC in osteoclast actin organization. Visualization of WT osteoclasts on bone slices by transmission electron microscopy showed the formation of the ruffled border which is confined by an actin-rich sealing zone (Fig. 3A, left), both characteristic morphological features of polarized osteoclasts required for bone resorption^9^. In contrast, *Hem1*-deficient osteoclasts lack the characteristic finger-like foldings of the ruffled membrane and instead display a homogenous and bulky structure (Fig. 3A, right). Moreover, in WT osteoclasts numerous vesicular structures were visible close to the ruffled-border apical membrane (as indicated by yellow arrows), while this was absent in Hem1-/- osteoclasts. The large number of vesicles in this region suggests a functional transport to the ruffled border and the release of lysosomal enzymes, which seems to be impaired in the Hem1-/- osteoclasts.

Since actin ring formation is essential for osteoclast polarization and efficient bone resorption^28,40^, we next sought whether *Hem1*^*-/-*^ osteoclasts are able to establish podosomes and actin rings. As shown in Fig. 3B, both control and *Hem1*^*-/-*^ osteoclast were able to establish actin rings. Interestingly, in case of *Hem1*^*-/-*^ osteoclasts grown on uncoated culture plates not completely closed actin rings were observed frequently (Fig. 3B, upper panel). *Hem1*^*-/-*^ osteoclasts seeded on CaPO_4_^2-^ displayed closed actin rings that appeared similar to that of WT osteoclasts (Fig. 3B, lower panel). This is consistent with the fact that the formation of podosomes and actin rings on bone surfaces is WASP- and not Hem1-dependent^29^. However, further analysis of immunofluorescent labelling of F-actin showed an abnormal podosome pattern in actin rings of *Hem1*-deficient osteoclasts differentiated on both, plastic and CaPO_4_^2-^ (Fig. 3C). Quantitative analysis revealed that about 86% of the actin rings of Hem1-/- osteoclasts but only 8% of the actin rings of WT osteoclasts lack a clear border between the sealing zone and the cell periphery (Fig. 3D). In line with the absent ruffled border, elevated F-actin and diffusely distributed podosomes were also present inside the actin ring of *Hem1*^*-/-*^ osteoclasts, resembling of the sealing zone-structures inside the actin ring (Fig. 3D). This observation of a disturbed ruffled border formation fits with previous results demonstrating that Hem1 is essential for functional phagocytosis in macrophages and required for the formation of a phagocytic cup^25^.

Besides disturbed ruffled border formation, we found that *Hem1*-deficient osteoclasts contained a lower intracellular pH (Fig. 3E), which potentially arises from accumulation of acidic vesicles unable to fuse with the ruffled border to lower the pH of the resorption lacuna.

In order to confirm an impaired vesicle transport in Hem1-deficient osteoclasts, immunofluorescence staining of cathepsin K and Rab7 in *WT* and *Hem1*^*-/-*^ osteoclasts was performed. On plastic, both *WT* and *Hem1*^*-/-*^ osteoclasts displayed an isolated and scattered intracellular distribution of cathepsin K whereas osteoclasts cultured on CaP showed a rather uniform intracellular distribution of cathepsin K. However, no differences could be observed between WT and *Hem1*^*-/-*^ osteoclasts (Fig. S3).

**Figure 4:**
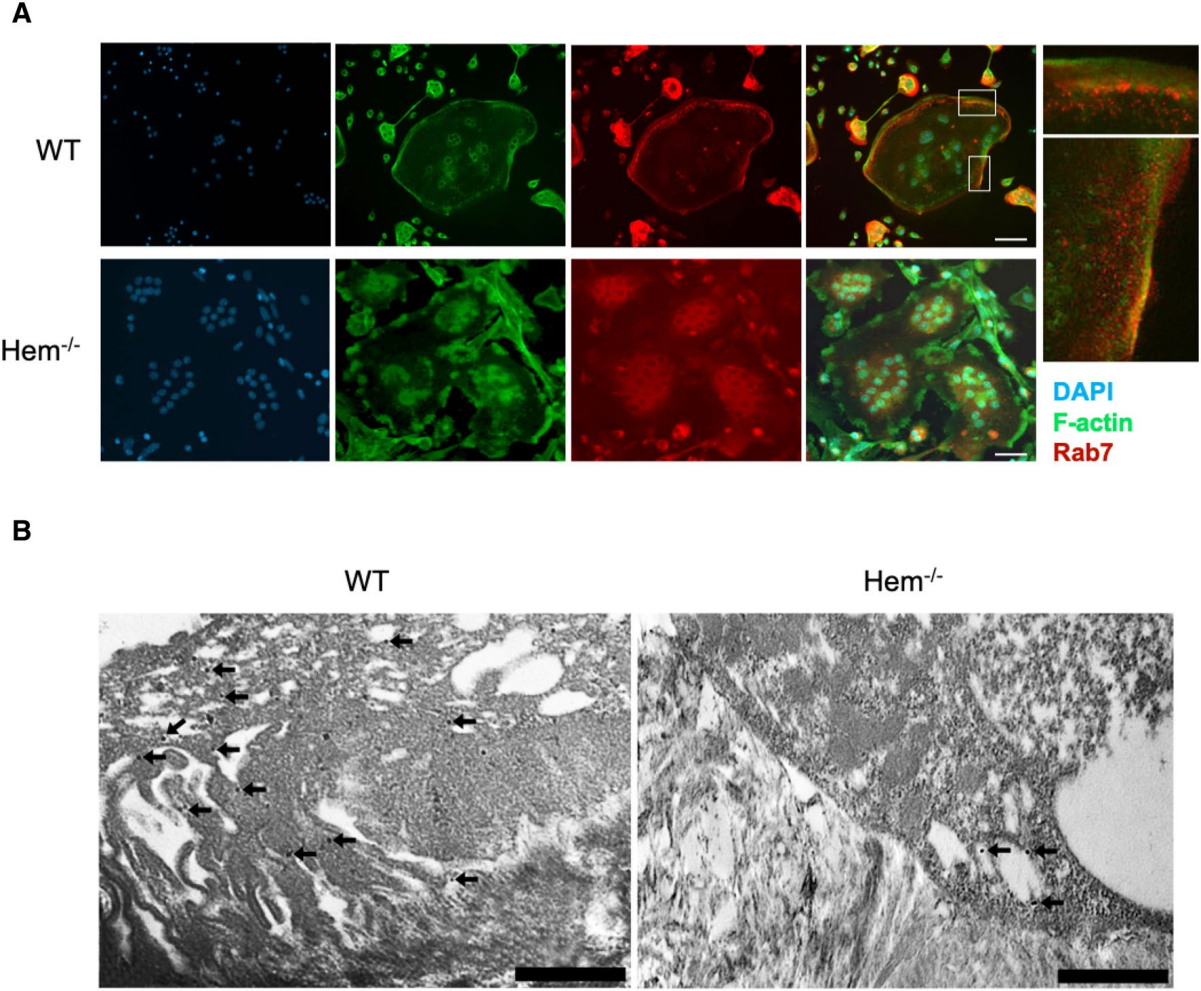
Distribution of Rab7 in *WT* and *Hem1*^*-/-*^ osteoclasts. (**A**) Representative immunofluorescent staining of Rab7 in *WT* and *Hem1*^*-/-*^ osteoclasts cultured on plastic. Scale bar 100 µm. (**B**) Immunogold labeling of Rab7 on cross-sections of *WT* and *Hem1*^*-/-*^ osteoclasts cultured on bovine bone slices. Black arrows indicate Rab7 antibodies coupled to gold beads. Scale bar 5 µm.

In contrast, distribution of Rab7 was different in *WT* and *Hem1*^*-/-*^ osteoclasts. While *WT* osteoclasts showed a clear localization of Rab7 at the cell periphery, Rab7 was rather localized in the center of *Hem1*-deleted osteoclasts (Fig. 4A). Since differences were already apparent in osteoclasts cultured on plastic, we performed further detailed studies on resorbing osteoclasts using immunogold labelling. Most interestingly, lokalisation of Rab7 at the ruffled-border indicates proper vesicle transport and fusion in *WT* osteoclasts whereas the lack of this late endosomal marker points to an impaired transport and fusion in the Hem^-/-^ osteoclasts (Fig. 4B).

### *Hem1* deletion in osteoclasts is accountable for osteoclast dysfunction leading to high bone mass phenotype

**Figure 5:**
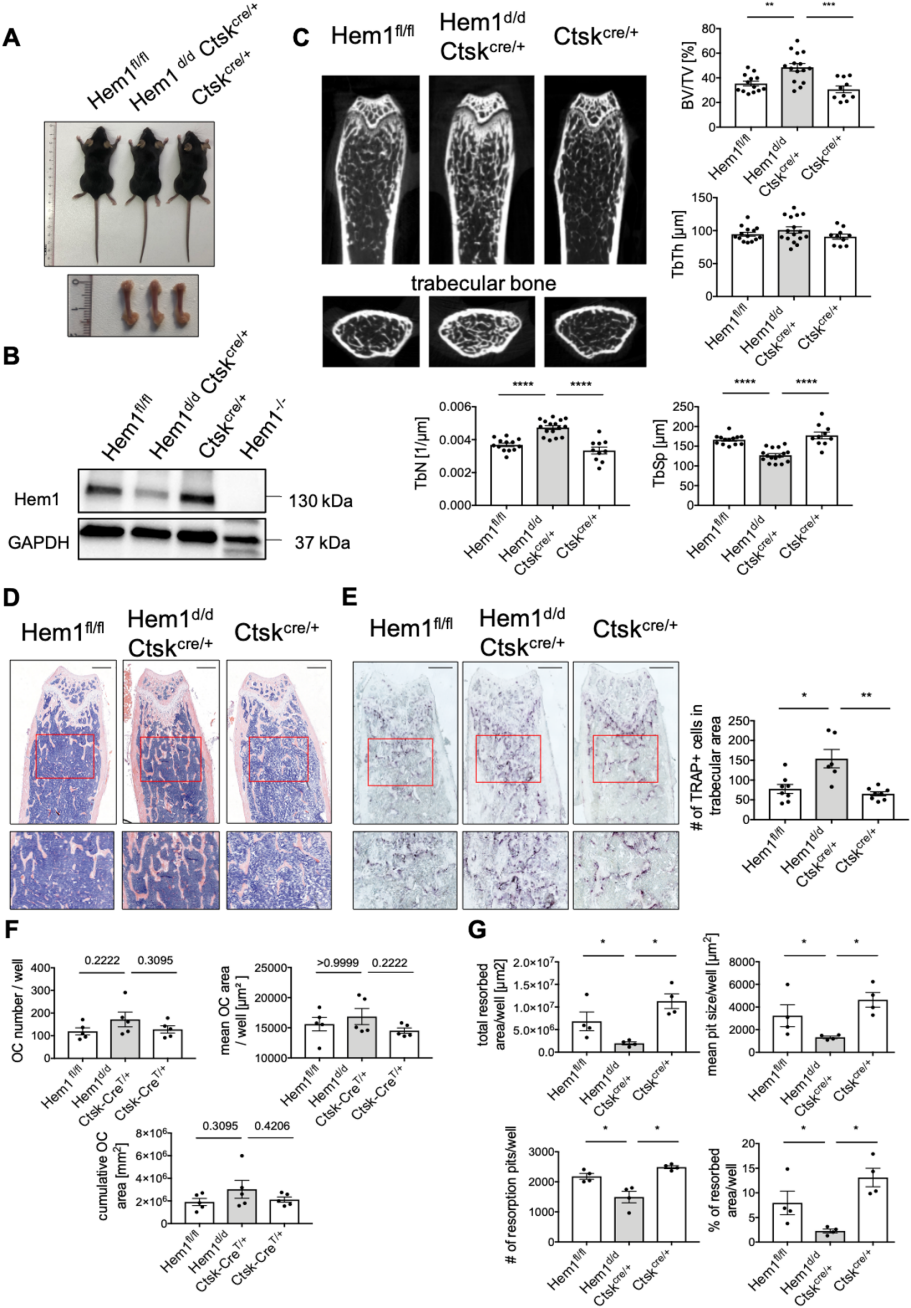
Hem1 deletion in osteoclasts is accountable for osteoclast dysfunction leading to high bone mass. (**A**) Osteoclast-specific deletion of Hem1 does not affect overall mouse phenotype. (**B**) Immunoblotting of Hem1 from Hem1^fl/fl^, Hem1^d/d^Ctsk^cre/+^, Ctsk^cre/+^ osteoclast lysates, representative images from n = 5 independent experiments. (**C**) Representative µCT cross sections of distal femora of Hem1^fl/fl^, Hem1^d/d^Ctsk^cre/+^, Ctsk^cre/+^ animals. Statistical analysis of trabecular bone volume to tissue volume (BV/TV,) trabecular thickness (Tb.Th.), trabecular number (Tb.N.) and trabecular separation (Tb.Sp.) of trabecular bone from 10–12 weeks old Hem1^fl/fl^, Hem1^d/d^Ctsk^cre/+^, Ctsk^cre/+^ male mice. ** = *P* 0.0022 (BV/TV), *** *P* = 0.0004 (BV/TV), **** *P* < 0.0001 (Tb.N, Tb.Sp.), two-tailed Mann–Whitney *U* test. **(D)** Representative H&E staining of distal femora from Hem1^fl/fl^, Hem1^d/d^Ctsk^cre/+^, Ctsk^cre/+^ mice, red box represents magnified area. (**E**) Left: representative TRAP staining of distal femora from Hem1^fl/fl^, Hem1^d/d^Ctsk^cre/+^, Ctsk^cre/+^ mice, red box represents magnified area. Right: Quantification of TRAP-positive osteoclasts within trabecular region from distal femora of Hem1^fl/fl^, Hem1^d/d^Ctsk^cre/+^, Ctsk^cre/+^ mice. * *P* = 0.02, ** *P* = 0.0013, two-tailed Mann–Whitney *U* test. All data presented as mean ± SEM. (**F**) Quantification of *in-vitro* osteoclast differentiation TRAP-assay of Hem1^fl/fl^, Hem1^d/d^Ctsk^cre/+^, Ctsk^cre/+^osteoclasts. (**G**) Quantification of resorption pit formation of Hem1^fl/fl^, Hem1^d/d^Ctsk^cre/+^, Ctsk^cre/+^ osteoclasts cultured on CaP-coated substrate. * *P* = 0.0286, two-tailed Mann–Whitney *U* test. All data presented as mean ± SEM.

In order to investigate the effect of *Hem1*-deficiency exclusively in mature osteoclasts committed to bone resorption, we generated *Hem1*^*d/d*^*-Ctsk*^*cre/*+^ mice^41^. In contrast to *Hem1*^*-/-*^ mice, *Hem1*^*d/d*^*-Ctsk*^*cre*+^ mice showed a slightly attenuated phenotype compared to *Hem1*^*fl/fl*^ and *Ctsk*^*cre/*+^ control animals (Fig. 5A). However, after 5 to 8 days of M-CSF and RANKL stimulation an apparent reduction of Hem1 was detectable in *Hem1*^*d/d*^*-Ctsk*^*cre/*+^ bone marrow-derived osteoclasts in vitro (Fig. 5B). Moreover, µCT analysis showed that osteoclast-specific deletion of *Hem1* led to an atypical bone phenotype (Fig. 5C; Fig. S2), resembling features of osteopetrosis and further stressing the role of Hem1 and thus WRC in osteoclast function. In line with total deletion of *Hem1*, *Hem1*^*d/d*^*-Ctsk*^*cre/*+^ mice developed higher trabecular BV/TV ratio (48%) as their control *Hem1*^*fl/fl*^ and *Ctsk*^*cre/*+^ littermates (35% and 30%, respectively), increased trabecular numbers (0.0047/µm vs 0.0037/µm and 0.0033/µm) and decreased trabecular separation (127 µm vs 166 µm and 177 µm). Interestingly, trabecular thickness was significantly increased in femora of female mice (Fig. S2) while unaltered in femora of male mice (Fig. 5C). Elevated trabecular bone mass in *Hem1*^*d/d*^*-Ctsk*^*cre/*+^ was further confirmed by H&E staining (Fig. 5D). Notably, higher osteoclast numbers by about 2.2-fold could be detected in femoral sections from *Hem1*^*d/d*^*-Ctsk*^*cre/*+^ mice (Fig. 5E). Number and size of in vitro differentiated *Hem1*^*d/d*^*-Ctsk*^*cre/*+^ osteoclasts were slightly increased (Fig. 5F) while their resorption ability was significantly diminished (Fig. 5G).

Taken together our results demonstrate for the first time an important function of the WRC subunit Hem1 in osteoclasts. Although Hem1 and the WRC appear not to be crucial for osteoclast differentiation, they are essential for osteoclasts to form a functional actin ring and the ruffled border in order to perform efficient bone resorption.

## Discussion

Bone remodelling is a tightly regulated process demanding a balance between bone formation and bone resorption. On one hand, some pathological conditions such as rheumatoid arthritis, osteoporosis or cancer-induced osteolytic lesions are characterized by pathological bone loss due to increased osteoclast numbers and/or osteoclast activity, while on the other hand, defects in osteoclastic differentiation or resorption are manifesting as osteopetrosis^1,2^. This highlights the importance of understanding the detailed molecular mechanisms underlying the bone resorption process. This study makes an important contribution to the understanding of the resorption mechanism in osteoclasts by identifying Hem1 as an essential player in bone remodelling. We demonstrated that upon Hem1 loss osteoclasts fail to proper organize their actin ring and ruffled border resulting in severely impaired bone resorption.

During bone resorption, osteoclasts undergo a successive reconstruction of membrane domains creating three distinct and specialized domains. Initially, osteoclasts establish an actin-rich sealing zone to confine and limit the site of bone resorption. In a second step, a high number of endosomal vesicles fuses with the bone-apposed plasma membrane forming the ruffled border within the sealing zone. From this highly convoluted membrane, protons and proteases are secreted into the extracellular space in order to demineralize the organic bone matrix and degrade collagen. Eventually, clathrin-mediated endocytosis facilitates the internalization of bone matrix fragments and transcytosis to the functional secretory domain where the degradation products are released into the extracellular space. These enormous structural changes of the osteoclast’s resorption machinery require engagement of the actin cytoskeleton^8–13^. The organization of the actin network in osteoclasts is particularly exceptional compared to that in other cell types and is controlled by GTPases of the Rho family, of which Rho, Rac and Cdc42 are the most studied in the context of bone resorption. It has been shown that Rho in osteoclasts is required for podosome stability, sealing zone formation and osteoclast polarization^12,42–46^.

Rac and Cdc42 are both known to control Arp2/3-complex-dependent branched actin polymerization through activation of their downstream effector WRC and (N)-WASP, respectively^47,48^. It has been established that both GTPases play an important role in osteoclasts and although individual deletion of both small GTPases results in osteopetrosis their function in osteoclasts is notably different^49,50^. Studies from Cdc42 loss-of-function mice provided evidence that besides positively regulating osteoclast precursor proliferation, differentiation and survival, Cdc42 induced osteoclast polarization while sealing zone formation itself was unaffected^49^. In a previous study, osteoclasts from WASP-null mice failed to form podosomes and consequently sealing zones resulting in reduced resorption capacity in vitro. In vivo, a resorption defect was only observed under resorptive conditions caused by ovariectomy but not under physiological conditions suggesting the existence of compensatory mechanisms in the absence of WASP^29^.

In contrast to Cdc42 loss-of-function or *WASP* deletion, global and osteoclast-specific deletion of *Hem1* led to severely reduced resorption in vitro as well as in vivo leading to an osteopetrosis-like phenotype. Our observations reflect to some extent the phenotype observed in *Rac1/Rac2* double knockout mice^50^. Rac stimulates Arp2/3-mediated branched actin polymerization required for lamellipodia formation and membrane ruffles through its downstream effector the WRC^51^ but also through p21-activated kinase (PAK)^47^. Unlike observed in the *Cdc42* knockout mice^49^, osteoclastogenesis was neither impaired in *Hem1* knockout mice nor in the *Rac1/Rac2*- knockout mice^50^, allowing the increased bone mineral content to be attributed to a resorption defect of osteoclasts. This is in some contrast to a recent study by Wang et al. who also observed a resorption defect due to Hem1 deletion, but also a reduced osteoclast differentiation due to impaired fusion in vitro*.* However, consistent with our observations, their Hem1-deleted mice showed an osteopetrotic phenotype with an increased number of osteoclasts, which is not indicative of reduced osteoclast differentiation and suggests that the contrasting in vitro results are rather caused by different experimental settings^30^.

Indeed, in line with our observation, osteoclast-specific deletion of *Rac1/Rac2* results in abnormal high numbers of osteoclasts in vivo and in vitro^50^, further underlining that Hem1-deletion does not disturb osteoclast precursor proliferation and differentiation. This is further confirmed by the increase in expression of osteoclastic markers during osteoclastogenesis of *Hem1*-deleted bone marrow cells.

Both, Rac1/Rac2-deficient as well as Hem1-deficient osteoclasts lack key characteristics of the resorption machinery e.g. Rac1/Rac2-deficient osteoclasts failed to form a sealing zone^50^. The fact that Hem1-deficient osteoclasts still form sealing zones while ruffled borders are absent leads to the assumption that formation of the sealing zone is regulated by Rac without involvement of Hem1/the WRC while formation of the ruffled border is dependent on Rac-dependent Hem1/WRC activity. This is supported by the observation that treatment of osteoclasts with either Rac1 or Rac2 antibodies led to severe disruption of sealing zones in osteoclasts resulting in accumulation of actin patches^52^.

We observed an accumulation of acidic vesicles in Hem 1-deficient osteoclast, suggesting that fusion of vesicles with the ruffled border is impaired. However, and quite interesting, a mechanistic link between Hem1 or the WRC and endosome fusion at the ventral osteoclast membrane has so far not been established. In this regard, Rac1 has been found to co-localize with Rab7 at the fusion zone of the ruffled border in osteoclasts reflecting a potential role in late endosomal trafficking and regulation of ruffled border formation^53^. The ruffled border allows further subdivision into two functional domains: the central uptake zone and peripheral fusion zone. Endocytosis of degradation products takes place in the centre where tubulin, clathrin and clathrin-associated coat proteins were found to accumulate while these are absent at the periphery of the ruffled border. On the other hand, at this peripheral domain of the ruffled border, which is adjacent to the actin ring, acidic vesicles containing Cathepsin K fuse with the membrane^11^. This region is devoid of microtubules but also shows actin localization as well as co-localization of Rac1 and Rab7 with actin^11,53^. Hence, this observation leads to the hypothesis that Rac1-WRC-mediated actin-branching could play a role in assembling the fusion zone within the peripheral ruffled border of osteoclasts. Taken all this into account, we assume that in the absence of Hem1 and the WRC vesicles accumulated within the cell but their fusion with the ventral membrane and consequently the release of their acidic content and proteases is inhibited. This assumption is supported by the absence of Rab7 at the apical membrane of Hem1 deficient osteoclasts in contrast to a prominent localization of Rab7 in the ruffled border of *WT* osteoclasts. The absence Rab7 and the absence of the ruffled border clearly indicates impaired vesicle transport and fusion in Hem^-/-^ osteoclasts. However, whether Rac1 is participating or to which extent Hem1 and the WRC is involved in this process requires further investigation.

It is evident that organization of the podosomes within the actin ring is disturbed in the absence of *Hem1*. The ruffled border in *Hem1*^*-/-*^ osteoclasts resembles structures of the sealing zone while the characteristic structures, the finger-like infoldings, are completely missing. This observation from TEM images is in line with our finding from immunofluorescent staining where we observed a high number of podosomes within the ruffled border of *Hem1*^*-/-*^ osteoclasts while WT osteoclasts are devoid of podosomes within this region. A recent observation in Hem1-deficient macrophages has shown that while integrin expression was slightly increased they show defects in integrin activation leading to the loss of lamellipodia and impaired phagocytic cup formation, processes that are dependent on actin branching^25^. In this regard, osteoclasts express αvβ3 integrin which is required for osteoclast polarity and cytoskeletal organization and inhibition of αvβ3 integrin or deletion of integrin β3 has been shown to cause osteoclast dysfunction and defective ruffled border formation resulting in osteopetrosis^5,35,36^. Since it has been demonstrated that in resorbing osteoclasts activated αvβ3-integrins are mainly localized at the ruffled border^54,55^, it may be that Hem1 and WRC play a role in activating αvβ3-integrin, which is required at the ruffled-border membrane and ensures proper resorption. Future research is needed to investigate the precise molecular action of Hem1 and the WRC in the context of sealing zone organization, ruffled border formation and osteoclastic bone resorption. The present study identifies Hem1 and the WRC as a crucial player in bone remodelling indispensable for a functional ruffled border and well resorbing osteoclasts.

## Materials and methods

### Animals

*Hem1*-deficient mice were generated as previously reported^31^. For osteoclast-specific *Hem1*-deletion, *Hem1*^*fl/fl*^ mice were crossed with *Ctsk*^*cre/*+^ mice^41^ to generate *Hem1*^*d/d*^*-Ctsk*^*cre/*+^ mice. Since *Ctsk*-Cre is a knock-in allele^41^, Ctsk^cre/+^ mice were bred as heterozygous in this study. Data was generated from age- and sex-matched male and female littermates at week 8–12. All strains were bred in the C57BL/6 background. Mice were held under SPF conditions in individually ventilated cages in groups on a 12:12-h light–dark cycle and access to food and water ad libitum. Mice were sacrificed at the age of 8–12 weeks by CO_2_ inhalation in compliance with the AVMA Guidelines for the Euthanasia of Animals. All animal procedures have been approved by the local ethics committee (Landesamt für Natur, Umwelt und Verbraucherschutz (84–02.04.2017.A143). All experiments were performed in compliance with the ARRIVE guidelines.

### Micro-CT imaging

Femora of 8–12 weeks old female and male mice were fixed overnight in 4% PFA followed by washing with PBS. Scans were performed 50 kV, 500 μA, 0.5 AL filter, 0.3° rotation steps and at a voxel resolution of 9 µm using Skyscan 1176 high resolution in vivo microtomography (Bruker). Reconstruction and analysis of bone parameters were performed according to international guidelines^56^ using manufacturers software (*Nrecon V1.7.4.2, CTan version 3.3.0r1401*; Bruker; www.bruker.com).

### Histomorphometrical analysis

Femora of 8–12 weeks old female and male were fixed overnight in 4% formalin and subsequently decalcified in 10% EDTA/1% PFA for two weeks, embedded into paraffin and sectioned into 5 µm slices. Sections were then stained with hematoxylin–eosin for discrimination between bone marrow, cartilage and bone. In vivo osteoclast number in distal femora was determined by TRAP (tartrate resistant acid phosphatase) staining using a TRAP Kit (#387A; Sigma Aldrich) according to manufacturer’s instructions.

### In vitro osteoclast generation

For isolation of mouse bone marrow derived macrophages (BMDM), femora and tibiae of 8–12 weeks old mice were aseptically removed and BMDM were flushed from the marrow cavity with PBS and cultured in α-MEM containing 10% FCS at 37 °C and 5% CO_2_. Differentiation of BMDM into osteoclasts was achieved by priming the cells with 30 ng/ml M-CSF (R&D) for two days followed by incubation in presence of 30 ng/ml MSCF and 50 ng/ml RANKL (R&D) for another five days. Osteoclast formation was visualized by TRAP staining.

### Osteoclast resorption assay

Osteoclastic resorption activity was measured by pit formation on calcium phosphate-coated 48-well plates according to manufacturer’s guidelines (Cosmo Bio). For this, cells were seeded and cultured as described above. Afterwards, cells were removed by treating the wells with 5% sodium hypochlorite and washed three times with PBS. Resorption pits were imaged using a Zeiss Observer.Z1 microscope (Zeiss) and number and area was measured with Zeiss AxioVision version 4.8. software (Zeiss; www.micro-shop.zeiss.com).

### Transmission electron microscopy

Osteoclasts were grown on 0,2 mm thick bovine bone slices (Boneslices.com) for 7 days as described above and subsequently fixed in 2% (v/v) formaldehyde and 2.5% (v/v) glutaraldehyde in 100 mM cacodylate buffer, pH 7.4, at 4 °C overnight. After washing in PBS, samples were postfixed in 0.5% (v/v) osmium tetroxide and 1% (w/v) potassium hexacyanoferrate (III) in 0.1 M cacodylate buffer for 2 h at 4 °C followed by washing with distilled water. After dehydration in an ascending ethanol series from 30 to 100% ethanol, samples were two times incubated in propylenoxide, each for 15 min. Finally, samples were embedded in Epon using embedding molds. Ultrathin sections were cut on an ultramicrotome and collected on copper grids. Electron micrographs were taken at a Phillips EM-410 electron microscope using imaging plates (Ditabis, Pforzheim, Germany).

For immunogold electron microscopy fixed samples were rinsed in PBS, dehydrated in ethanol up to 70%, and embedded in LR White embedding medium (London Resin Company, UK) according to the manufacturer´s instructions using UV light for polymerization. For immunogold electron microscopy, ultrathin sections were incubated with 100 mM glycin in PBS for 2 min, washed with PBS and blocked with 2% (w/v) BSA and 1% normal goat serum (Aurion) in PBS. Afterwards, ultrathin sections were incubated for 1 h at room temperature on drops of primary antibodies (Rab 7 (D95F2) from Cell Signaling) in PBS containing 1% (v/v) BSA-c (Aurion) and 0.025% (v/v) Tween 20. After washing with the same solution, ultrathin sections were incubated with secondary antibodies conjugated to 18 nm particles (Dianova). After washing with distilled water, ultrathin sections were negatively stained with 2% (w/v) uranyl acetate for 15 min. Electron micrographs were taken at 60 kV with a Veleta camera system in combination with the Radius software system version 2.2 (emsis, Münster, Germany, www.emsis.eu).

### Immunoblotting

After stimulation, whole cell lysates of osteoclasts were lysed in NP-40 buffer containing (150 mM sodium chloride, 1% NP-40, 50 mM Tris pH8) including protease and phosphatase inhibitor cocktail (#11,873,580,001, #P5726, #P004; Sigma Aldrich). Specific antibodies against the following proteins were detected by immunoblotting: cathepsin K (abcam, ab 19,027, 1:1000), integrin alpha-v (abcam, ab179475,1:1000), integrin beta-3 (Cell Signaling #4702,1:1000), DC-STAMP (sc-98769, 1:200), NFATc1 (sc-7294; both from Santa Cruz), Hem1 (Novus Bio, NBP2-13,643, 1:1000), WASP (Santa Cruz, sc-13139, 1:1000) and WAVE2 (Cell Signaling, #3659, 1:1000). For loading control purposes, membranes were stripped and re-probed for GAPDH (Cell Signaling, #3683, 1:2000).

### Immunofluorescence

To visualize acidic compartments, mature osteoclasts were incubated with LysoSensor™ Green DND-189 (Molecular Probes) at a concentration of 1 µM in cell culture media. After 30 min, cells were washed with fresh media twice and imaged using a Zeiss Observer.Z1 microscope and Zeiss AxioVision 4.8. software (Zeiss, www.micro-shop.zeiss.com). Analysis of the actin cytoskeleton was assessed by staining with Alexa488-conjugated phalloidin (Molecular Probes). Single slice images of osteoclasts were acquired using a Zeiss Observer.Z1 microscope and Zeiss AxioVision 4.8. software (Zeiss, www.micro-shop.zeiss.com).

For actin ring analysis Z-stacks covering the cell volume were acquired using 40× oil objective of a spinning disc confocal microscope (Zeiss Axio Observer Z1, Yokogawa CSU22 spinning disc module; Visitron Systems GmbH). Z-stacks were converted into maximum intensity z-projections using ImageJ/Fiji (Version 1.49p, National Institutes of Health, www.imagej.net) and F-actin intensity was measured throughout the actin ring.

For staining of Cathepsin K and Rab7, osteoclasts were washed in PBS and fixed with 4% PFA, pH 7.4 for 20 min. In a next step, the cells were washed and quenched with 100 mM NH_4_Cl. The cells were then permeabilized with 0.5% Tween 20 and subsequently washed with PBS. Next, the cells were then treated for one hour with blocking solution containing 10% normal horse serum (NHS). All samples were stained with a primary antibody and labelled secondary antibodies (Invitrogen). The nuclei were stained using 4′,6-diamidino-2-phenylindole (DAPI) (Invitrogen) and the cytoskeleton was stained with Rhodamin-Phalloidin (Invitrogen). We used following primary antibodies: anti-Rab7 (Cell Signaling) and anti-Cathepsin K (Abcam).

### Statistical analysis

Data are presented as means ± SEM. Statistical analysis was performed via the *GraphPad Prism* Software, version 7 (GraphPad Software Inc.; www.graphpad.com). Differences between groups were investigated for statistical significance using two-tailed Mann–Whitney *U* test. Paired data were statistically analysed by two-tailed Wilcoxon matched-pairs RANK test. A value of *P* < 0.05 was considered statistically significant.

### Supplementary Information

Supplementary Figures.

## Data Availability

All data needed to evaluate the conclusions in the paper are present in the paper and/or the Supplementary Materials.
