# Supplementary material for: Hem1 is essential for ruffled border formation in osteoclasts and efficient bone resorption
Source: Sci Rep. 2024 Apr 6;14:8109. doi: 10.1038/s41598-024-58110-x (PMC10998871; doi:10.1038/s41598-024-58110-x)

Hem1 is essential for ruffled border formation in osteoclasts and efficient bone resorption

Eugenie Werbenko,[1] David J. J. de Gorter,[1] Simon Kleimann,[1] Denise Beckmann,[1] Vanessa Waltereit-Kracke,[1] Julia Reinhardt,[1] Fabienne Geers,[1] Peter Paruzel,[1], Uwe Hansen,[1] Thomas Pap,[1] Theresia E.B. Stradal,[2] Berno Dankbar[1*]

*Corresponding author. Email: dankbarb@uni-muenster.de

This PDF file includes:

Fig. S1 Analysis of µCT parameters of female mice also revealed an osteopetrosis-like phenotype in Hem1$^{-/-}$

Fig. S2 Conditional deletion of Hem1 in female mice also confirmed higher trabecular bone mass

Fig. S3 Immunfluorescence staining of cathepsin K in osteoclasts cultured on plastic and CaP

Original blots

Fig. S1.

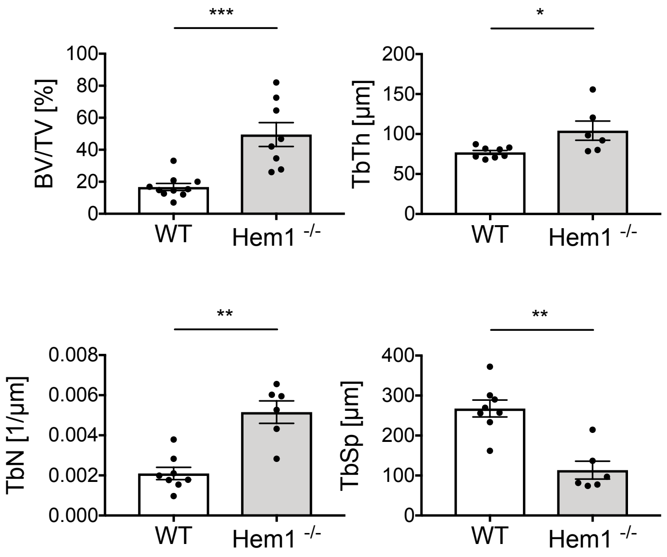

Analysis of µCT parameters of female mice also revealed an osteopetrosis-like phenotype in Hem1$^{-/-}$.

Statistical analysis of trabecular bone volume to tissue volume (BV/TV,) trabecular thickness (Tb.Th.), trabecular number (Tb.N.) and trabecular separation (Tb.Sp.) of trabecular bone from 8-12 weeks old WT and Hem1$^{-/-}$ female mice. *** P = 0.0002 (BV/TV), * = P 0.0426 (Tb.Th.), ** = P 0.0027 (Tb.N.), ** P = 0.0013 (Tb.Sp.), two-tailed Mann-Whitney U test.

Fig. S2.

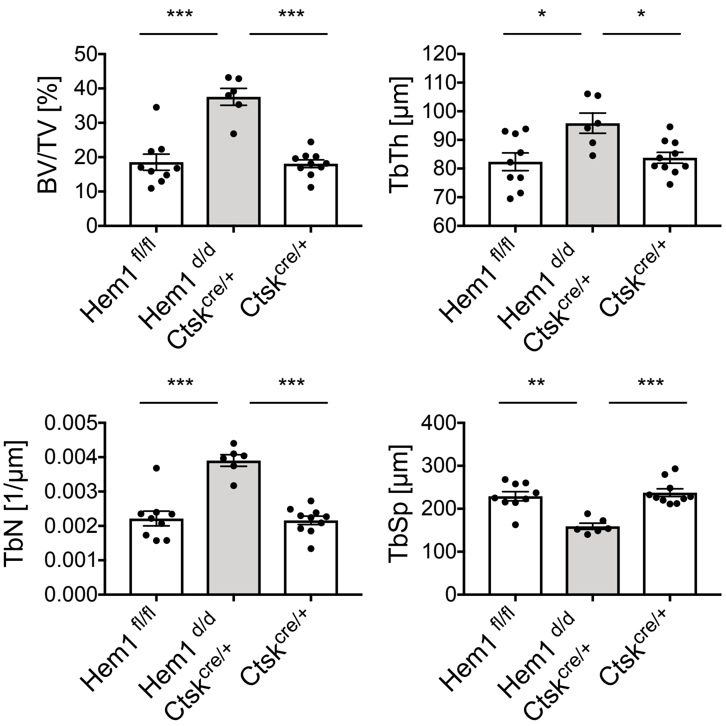

Conditional deletion of Hem1 in female mice also confirmed higher trabecular bone mass.

Statistical analysis of trabecular bone volume to tissue volume (BV/TV,) trabecular thickness (Tb.Th.), trabecular number (Tb.N.) and trabecular separation (Tb.Sp.) of trabecular bone from 10-12 weeks old Hem1$^{fl/fl}$, Hem1$^{d/d}$Ctsk$^{cre/+}$, Ctsk$^{cre/+}$ female mice. *** *P* = 0.0008 (BV/TV, Tb.N. Hem1$^{fl/fl}$ vs Hem1$^{d/d}$Ctsk$^{cre/+}$), *** *P* = 0.0002 (BV/TV, Tb.N., Tb.Sp. Hem1$^{d/d}$Ctsk$^{cre/+}$ vs Ctsk$^{cre/+}$), * *P* = 0.00176 (Tb.Th. Hem1$^{fl/fl}$ vs Hem1$^{d/d}$Ctsk$^{cre/+}$), * *P* = 0.0110 (Tb.Th. Hem1$^{d/d}$Ctsk$^{cre/+}$ vs Ctsk$^{cre/+}$), ** *P* = 0.0016 (Tb.Sp. Hem1$^{fl/fl}$ vs Hem1$^{d/d}$Ctsk$^{cre/+}$), two-tailed Mann-Whitney *U* test.

Fig. S3.

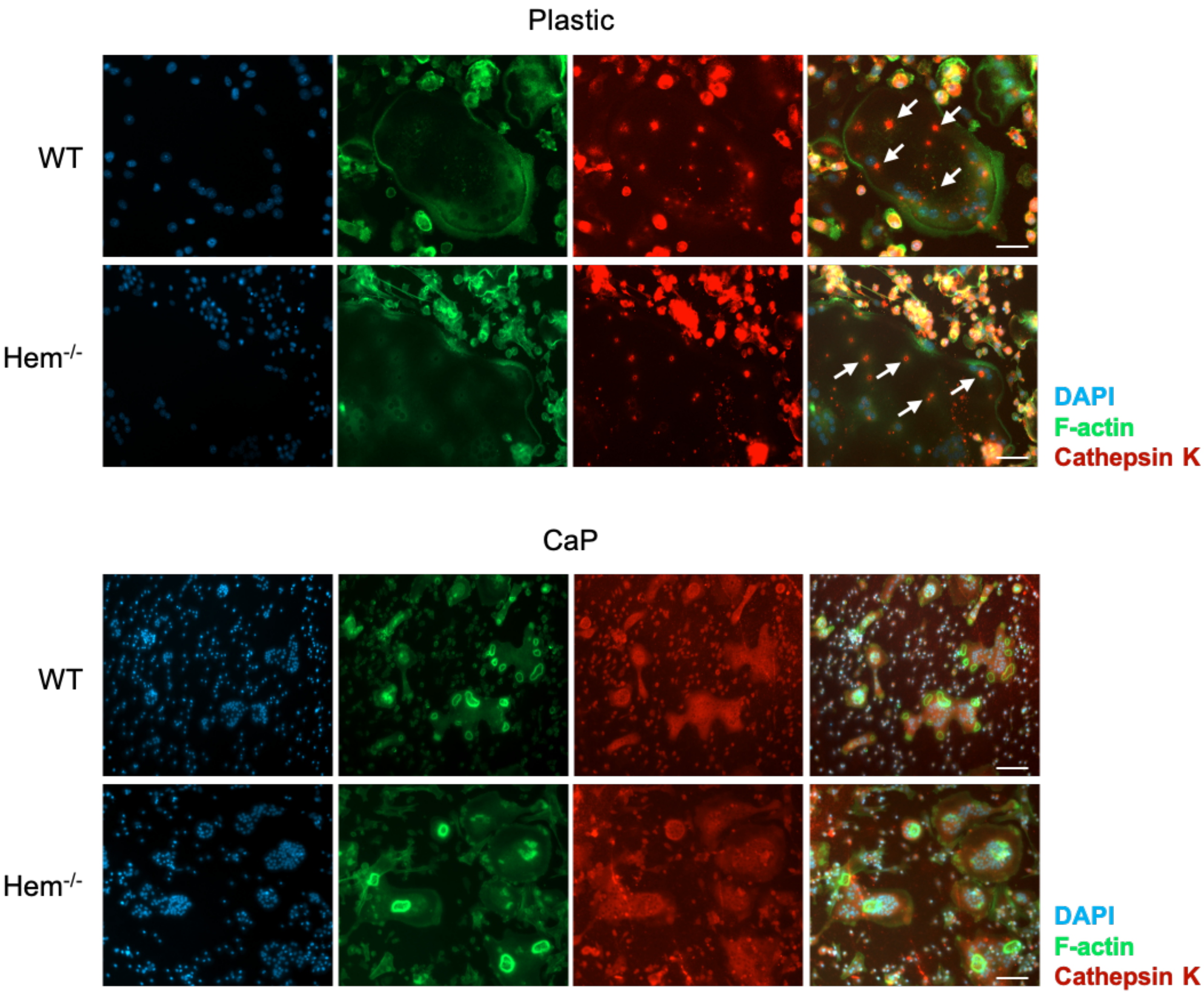

Immunofluorescence staining of cathepsin K in osteoclasts cultured on plastic and CaP.

Representative immunofluorescence staining of cathepsin K in wild-type and *Hem1*$^{-/-}$ osteoclasts cultured on plastic and CaP. White arrows in the upper rows indicate the isolated and scattered distribution of Cathepsin K in osteoclasts cultured on plastic, whereas the OC cultured on CaP showed a uniform intracellular distribution of cathepsin K. Scale bar 100μm (on Plastic), 50μm (on CaP).

Original blots

Fig. 2F

NFATc1

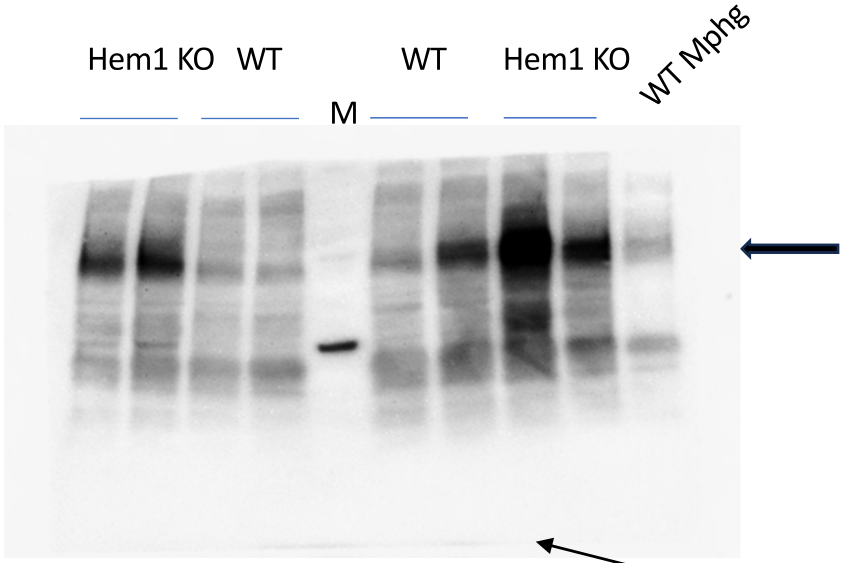

All images show full length membranes

Integrin aV

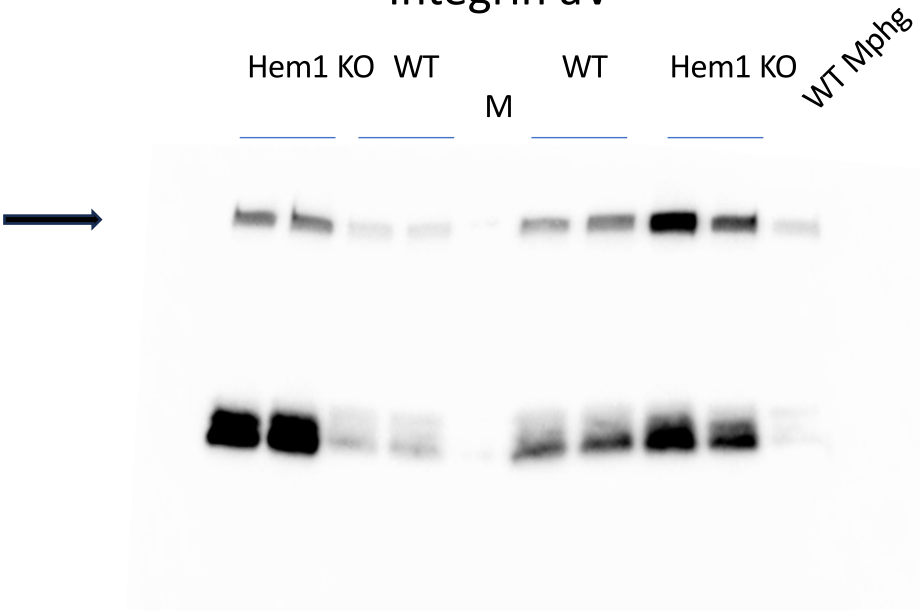

Same membrane as for NFATc1, stripped

We are not able to provide original images with longer exposure time to show membrane edges

GAPDH

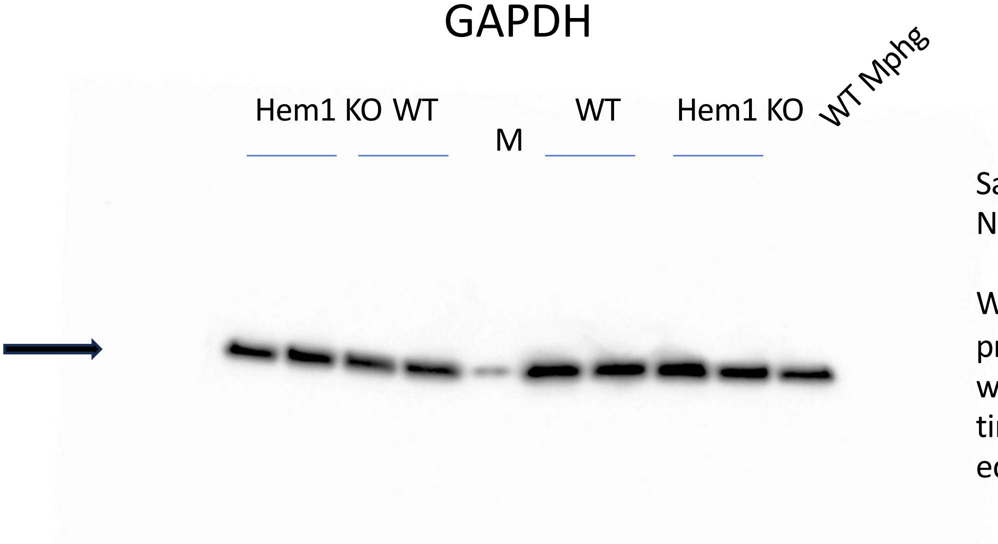

Same membrane as for NFATc1, stripped

We are not able to provide original images with longer exposure time to show membrane edges

DC-STAMP

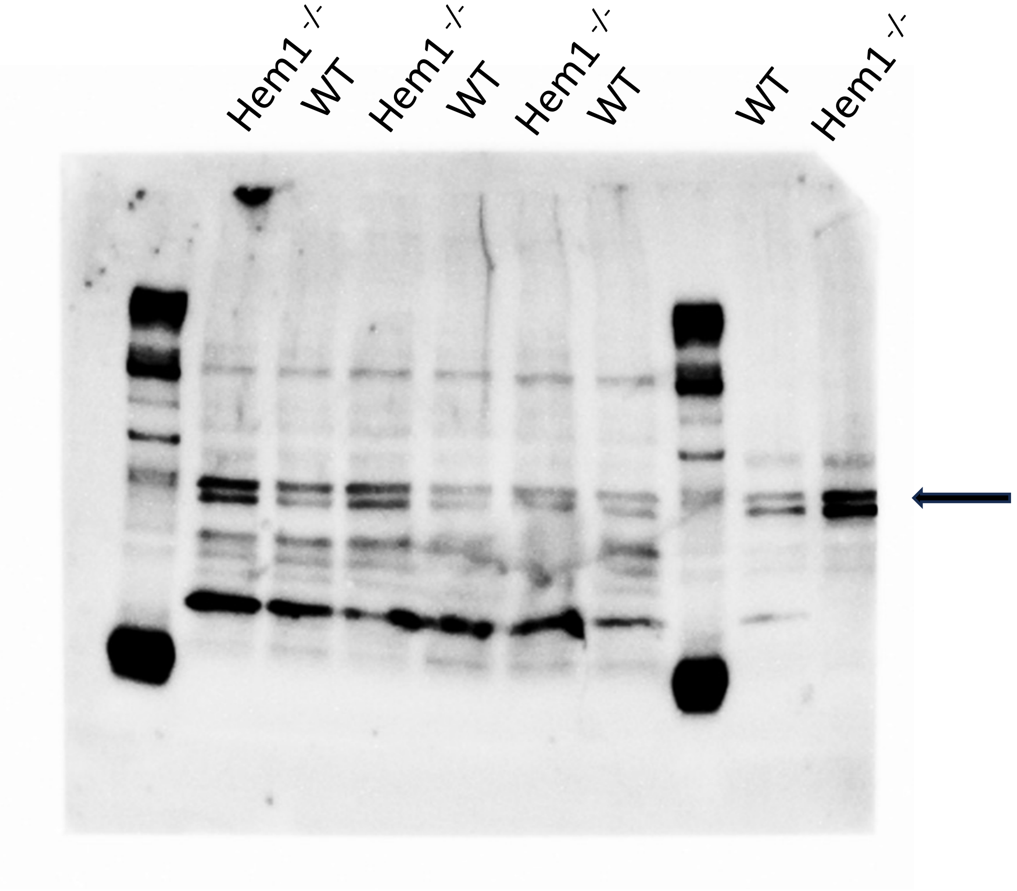

GAPDH

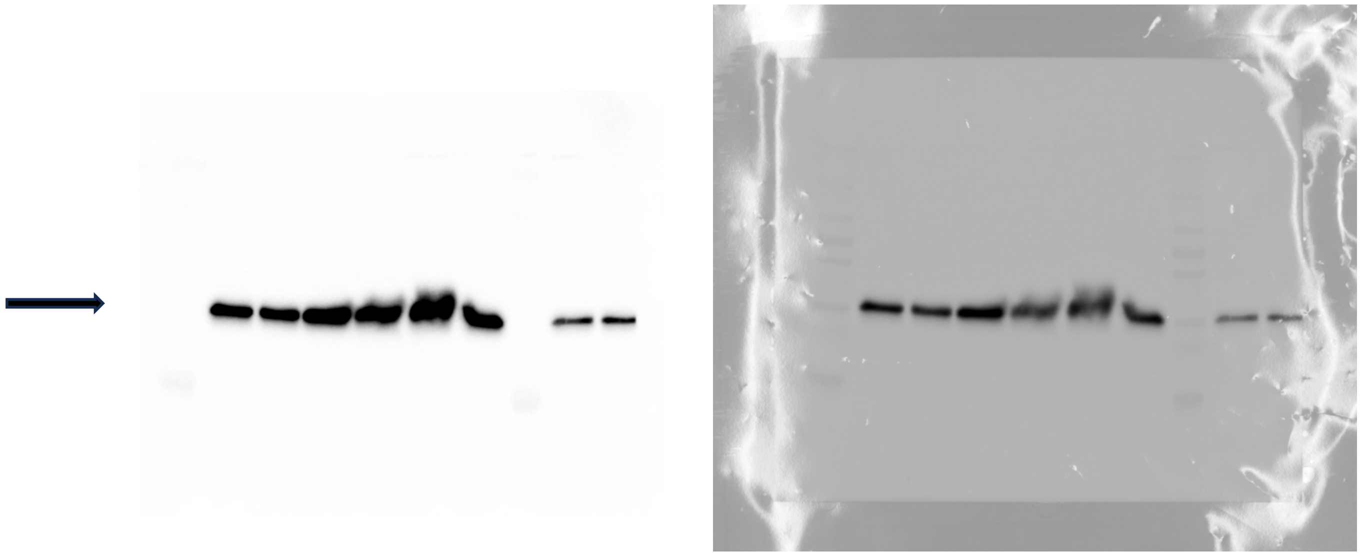

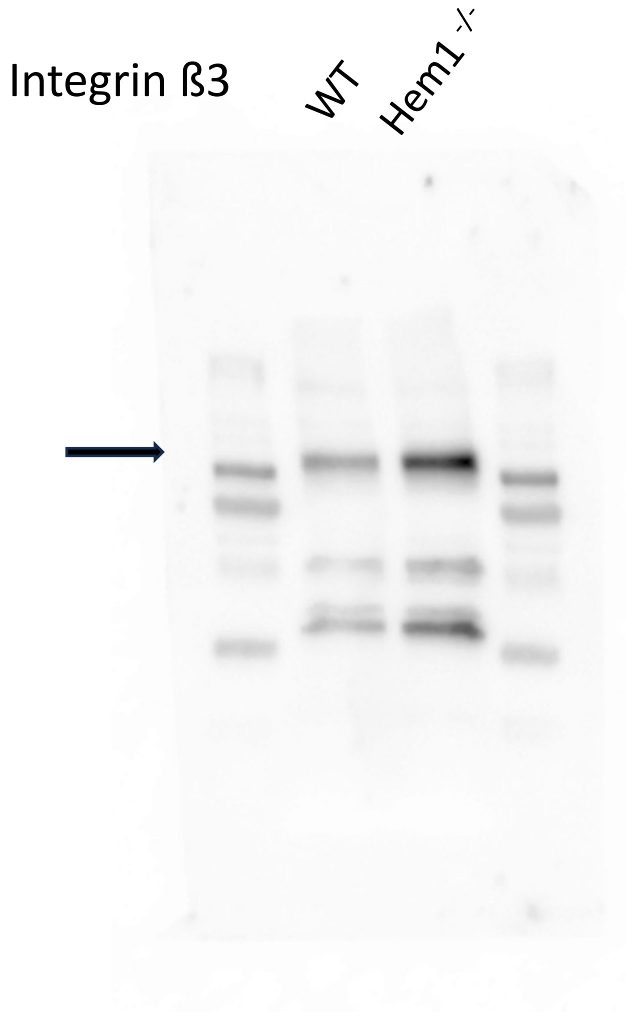
Integrin ß3
WT
Hem1 -/-

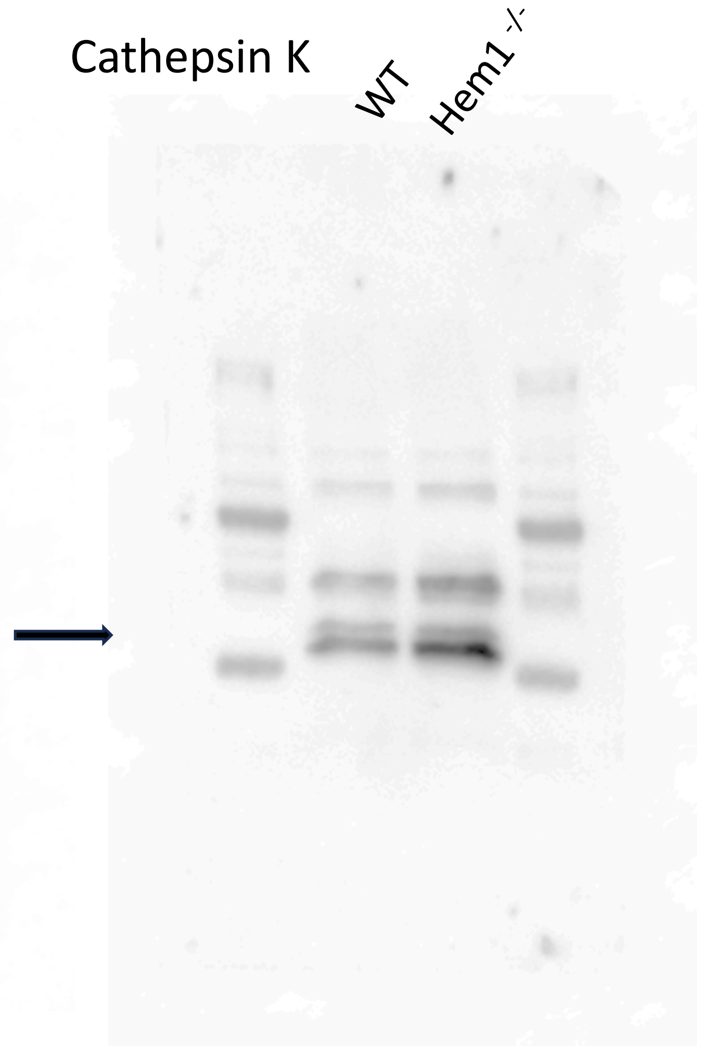
Cathepsin K
WT
Hem1 -/-

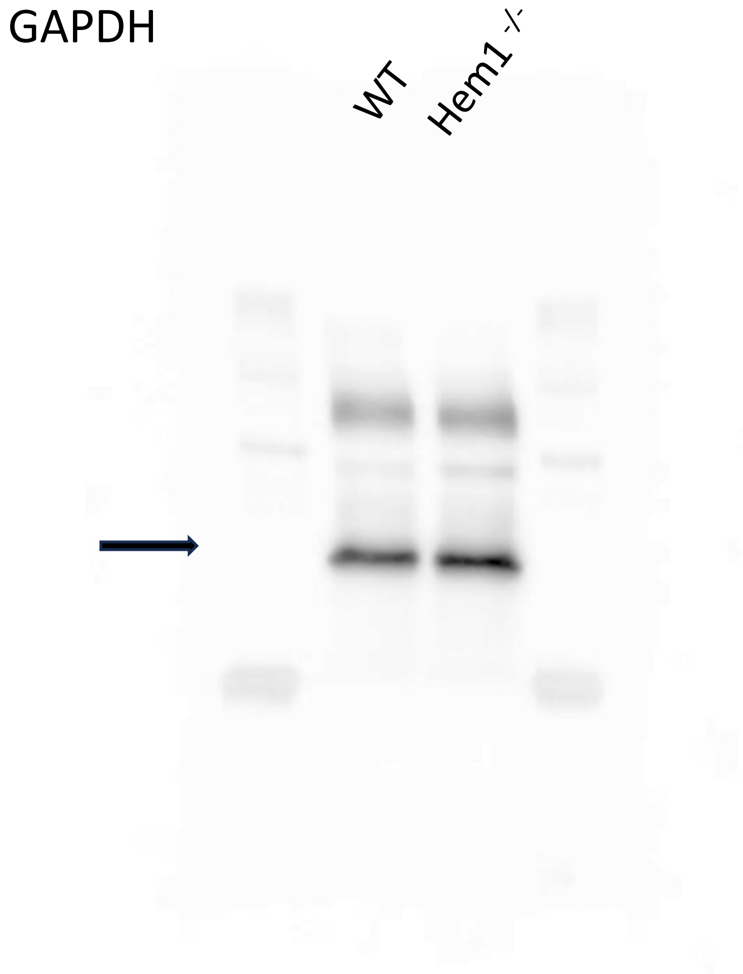
GAPDH
WT
Hem1 -/-

Fig. 2G

Hem1

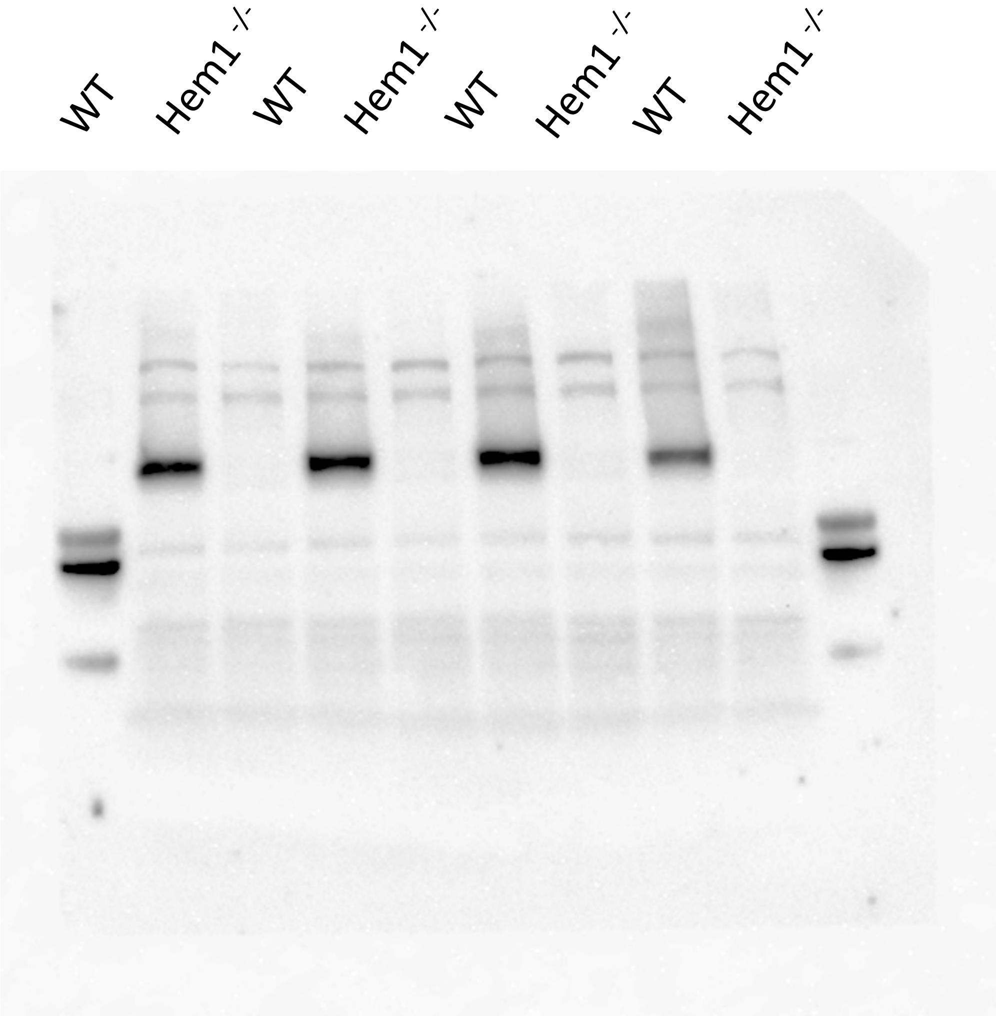

WAVE2

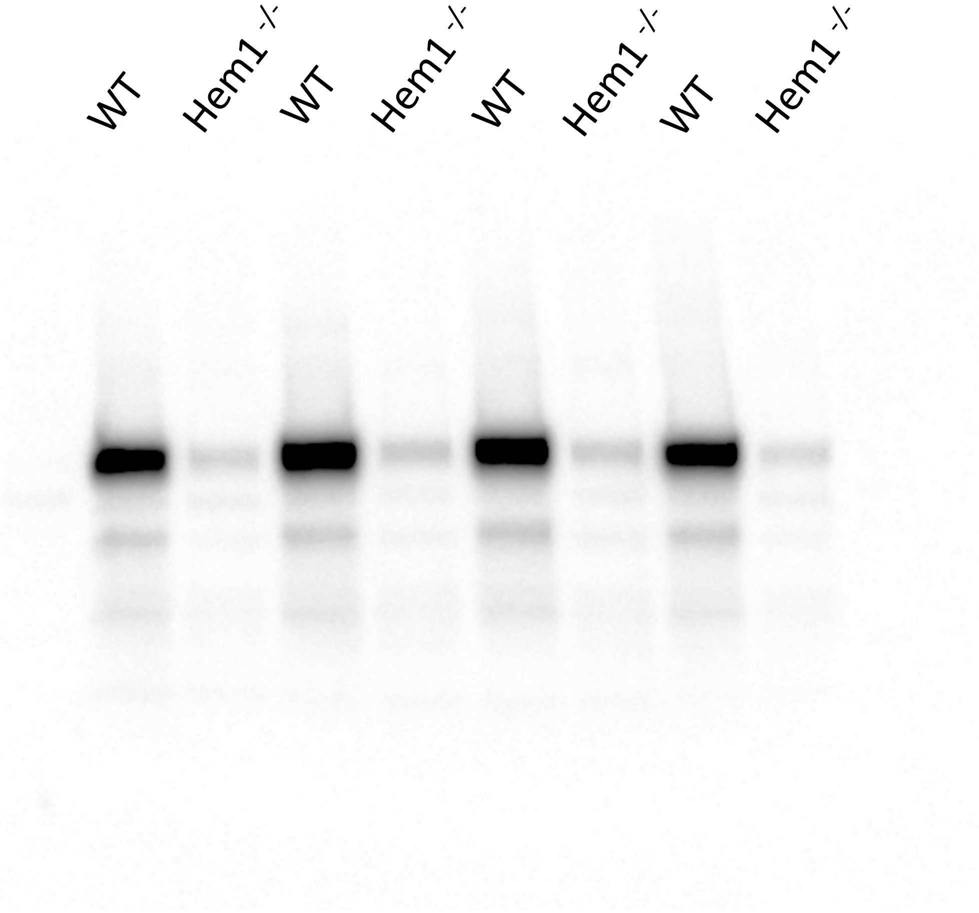

WASP

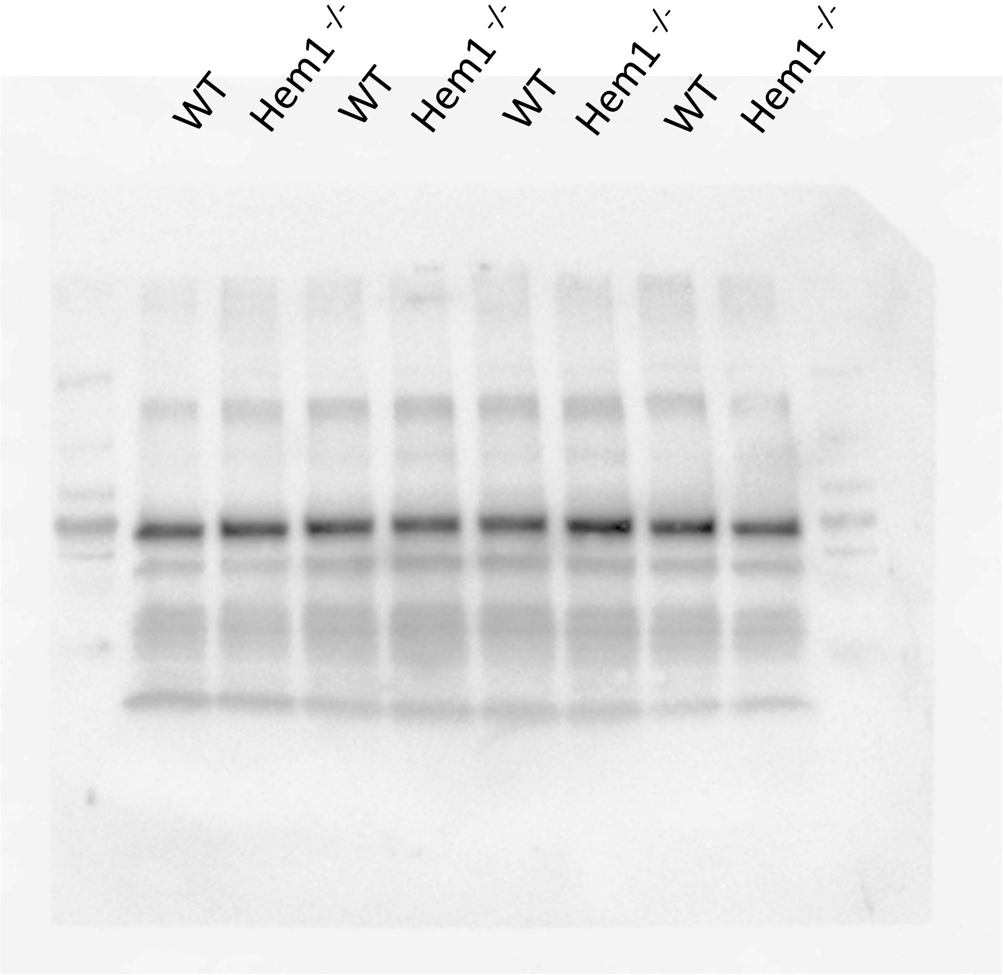

GAPDH

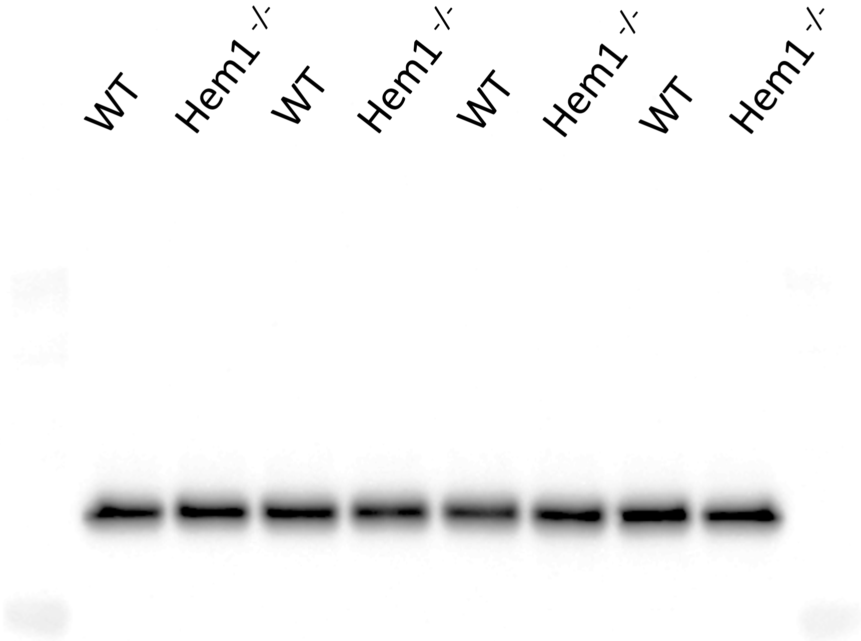

Same membrane as above, stripped

We are not able to provide original images with longer exposure time to show membrane edges

Fig. 5B

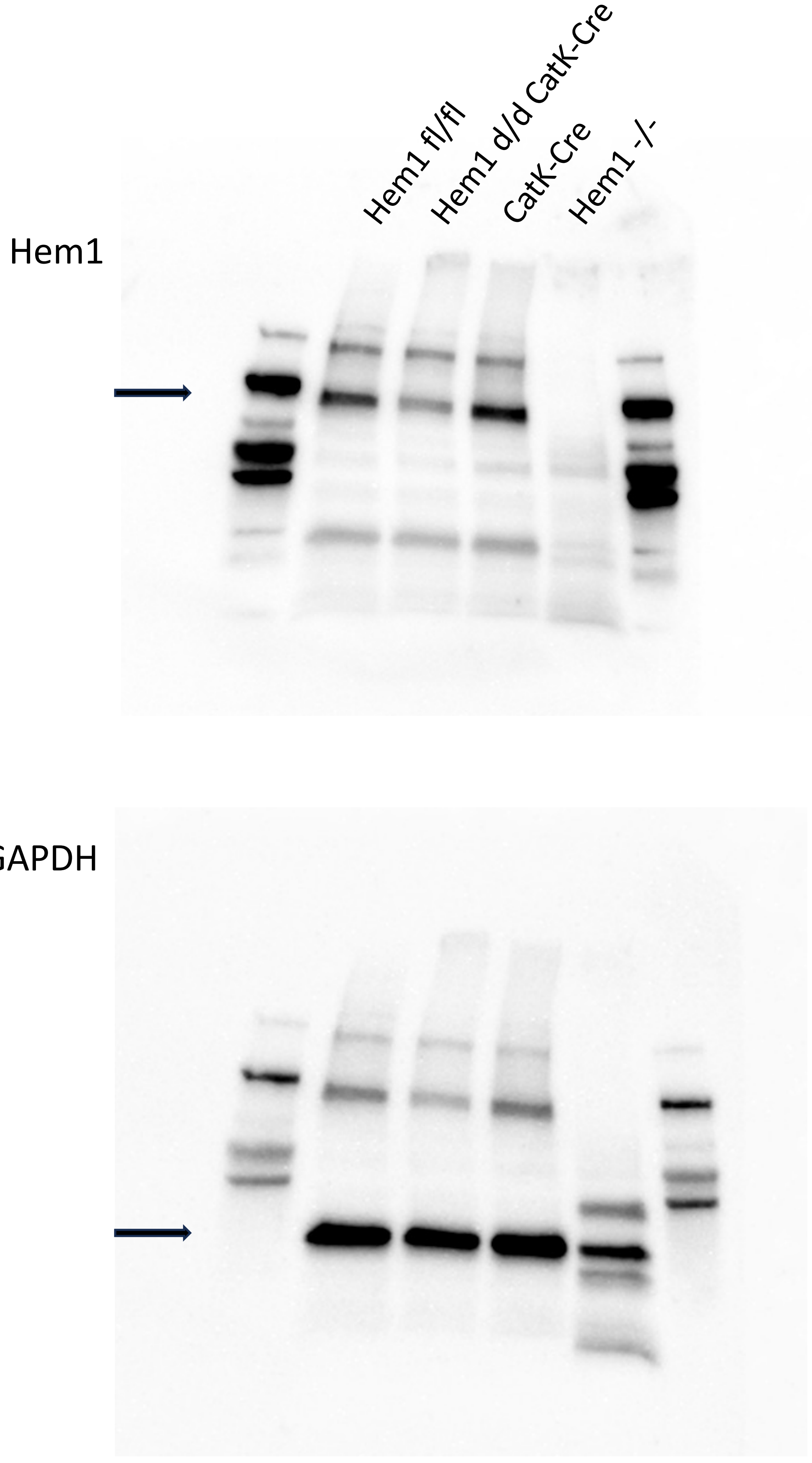

Supplement: Supplementary file 1 — Supplementary Figures. [file 41598_2024_58110_MOESM1_ESM.pdf]
